# Risk of HBV infection among male and female first-time blood donors born before and after the July 1986 HBV vaccination program in Taiwan

**DOI:** 10.1186/s12889-021-11846-x

**Published:** 2021-10-09

**Authors:** Hsuan-Hui Wang, Shu-Lung Sun, Rong-Chiou Jau, Disline Manli Tantoh, Shu-Yi Hsu, Oswald Ndi Nfor, Pei-Hsin Chen, Wen-Hsiu Liu, Jiunn-Liang Ko, Yung-Po Liaw

**Affiliations:** 1 Taichung Blood Center, Taiwan Blood Services Foundation, Taichung, Taiwan; 2 Taiwan Blood Services Foundation, Taipei, Taiwan; 3 Department of Medical Imaging, Chung Shan Medical University Hospital, Taichung, Taiwan; 4 Department of Public Health and Institute of Public Health, Chung Shan Medical University, No. 110, Sec. 1 Jianguo N. Rd, Taichung, 40201 Taiwan; 5 Institute of Medicine, Chung Shan Medical University, No. 110, Sec. 1 Jianguo N. Rd, Taichung, 40201 Taiwan; 6 Department of Medical Oncology and Chest Medicine, Chung Shan Medical University Hospital, Taichung, Taiwan; 7 Medical Imaging and Big Data Center, Chung Shan Medical University Hospital, Taichung, Taiwan

**Keywords:** HBV vaccination, Sex, First-time blood donor, Taiwan blood Services Foundation, HBV risk

## Abstract

**Background:**

In July 1984, Taiwan officially began a nationwide hepatitis B virus (HBV) vaccination program where only infants born to HBsAg-positive mothers were vaccinated free of charge until June 1986. However, from July 1986, all infants were vaccinated against HBV. The impact of the July 1986 HBV vaccination program on first-time blood donors has not been exhaustively studied. We, therefore, determined the risk of HBV among male and female first-time blood donors born before and after the July 1986 HBV vaccination program in Taiwan.

**Methods:**

Initially, we recruited 857,310 first-time blood donors whose data were collected between 2013 and 2018 from 5 blood donation centers in Taiwan. However, we excluded donors with incomplete and outlying data (*n* = 12,213) and those born between July 1984 and June 1986 (*n* = 21,054). The final study participants comprised 9118 HBV positive and 814,925 HBV negative individuals. We divided the participants into two birth cohorts (born before and after July 1986) and assumed that those born before July 1986 were not vaccinated at birth while those born after July 1986 were vaccinated.

**Results:**

The prevalence of HBV among those born before and after July 1986 was 4.53 and 0.25%, respectively. Individuals born after July 1986 had a lower risk of HBV than those born before July 1986. The adjusted odds ratio (OR), 95% confidence interval (CI) was 0.16, 0.13–0.19. Men had a higher risk of HBV than women (OR = 1.40, 95% CI = 1.34–1.47). The interaction between sex and birth date was significant (*p*-value = 0.0067). Stratification of participants by birth date revealed a higher risk of HBV in men compared to women in both birth cohorts. The OR, 95% CI was 1.47, 1.40–1.55 for those born before July 1986 but declined to 1.15, 1.02–1.29 for those born after July 1986.

**Conclusions:**

The risk of HBV was lower among those born after than those born before the July 1986 vaccination program. In both cohorts, the risk was high in men relative to women. The seemingly protective effect among those born after July 1986 was higher in women than men.

## Background

Viral hepatitis heightens the susceptibility to hepatocellular carcinoma (HCC) and chronic liver disease [1]. HBV infection is an important global public health problem, accounting for significant morbidity and mortality [2]. HBV infection remains an important transfusion-transmitted disease in Taiwan due to its high prevalence [3]. Universal vaccination programs have led to significant decreases in the proportion of people living with chronic HBV [4] as well as HBV-related morbidity and mortality [5, 6]. For example, young Chinese blood donors who were vaccinated before age 18 had a lower risk of HBV than those who were not vaccinated [7].

Before the introduction of mass HBV vaccination in Taiwan, approximately 15–20% of the general Taiwanese population tested positive for the HBV surface antigen (HBsAg), primarily because of vertical transmission [8–10]. In July 1984, Taiwan officially began a nationwide HBV vaccination program where only infants born to HBsAg-positive mothers were vaccinated free of charge until June 1986 [11]. However, from July 1986, all infants, irrespective of their mothers’ HBV status, were immunized against HBV with a 5 μg four-dose (administered at birth, 1, 2, and 12 months) plasma-derived vaccine [12]. Neonates born to highly infected mothers also received 0.5 mL of the HBV immunoglobulin within 24 h of birth [13]. The implementation of this nationwide vaccination program led to a substantial decline in HBV infection. For instance, the chronic HBV infection rate decreased from 9.7% among university students born before June 1974 to < 1% among those born after 1992 [14]. Moreover, the HBsAg-positive rate decreased from approximately 4.2% among university students born before 1984 to 0.6% among those born in 1999 [14].

HBV vaccination at birth could improve the safety of both donors and recipients during blood donation and transfusion. Several factors influence the effectiveness of vaccines. For instance, increasing age, male sex, and BMI ≥ 25 were associated with a lower immune response to HBV vaccination [15]. Undertaking a nucleic amplification test (NAT) for HBV during blood donation screening could improve safety during blood transfusion [7]. Blood group screening is a mandatory process in blood donation and transfusion because certain blood groups influence the pathogenesis of HBV and other transfusion-transmitted infections [16, 17]. Blood group antigens influence the transmission of viruses and other infectious agents by modulating the inflammatory and immune responses or by acting as ligands and receptors for such agents [18–21]. For instance, some ABO antigens are capable of blocking the binding of transfusion-transmitted disease-causing organisms to polysaccharides on the host’s cells while others are not  [22]. For HBV, Blood group O carriers have been found with a higher risk of infection than other blood group carriers [23].

A sufficient supply of safe blood to patients is an integral part of a country’s health care policy [24]. However, statistics from the Department of Household Registration, Ministry of Interior, indicate a shift in the population structure of Taiwan from a younger to an older generation [25]. This shift could adversely affect the demographic profile of first-time blood donors. It is, therefore, important to increase the proportion of first-time blood donors, particularly young people. The impact of the July 1986 national HBV vaccination program on first-time blood donors has not been systematically reported. The purpose of this study was to estimate the prevalence of and factors associated with HBV infection from 2013 to 2018 in male and female first-time blood donors who were born before and after the July 1986 HBV vaccination program in Taiwan.

## Materials and methods

Data were collected by the Taiwan Blood Services Foundation (TBSF) from 2013 to 2018. All volunteers filled and signed a blood donation registration form before donating blood. Some of the information included sex, height, weight, birth date, permanent address, and occupation. The eligibility criteria included age, 17 to 65 years old; male body weight and hemoglobin (Hb) level, ≥ 50 kg and ≥ 13.0 g/dL, respectively; female body weight and Hb level, ≥ 45 kg and ≥ 12.0 g/dL, respectively; systolic blood pressure (SBP), 90 to 160 mmHg; and diastolic blood pressure (DBP), 50 to 95 mmHg. These eligibility criteria correspond to the blood donation service requirements.

For allogeneic use, all donors underwent screening for the ABO blood type, rhesus (Rh) status, and transfusion-transmitted viral infection (TTVI) markers, such as HBsAg, HBV DNA, hepatitis C virus (HCV) RNA, human immunodeficiency virus (HIV) RNA, anti-HCV, anti-HIV, anti-HTLV-I/II antibodies to safeguard patients’ health. Any blood sample which tested positive for TTVIs or had abnormal levels of alanine aminotransferase (ALT) was discarded. Medications like etretinate, non-steroid anti-inflammation drug (NSAID), and antiplatelet drugs (e.g., aspirin) are potentially harmful to blood recipients or developing fetuses. So, all blood samples from donors taking such medications were discarded because such donors were considered high-risk individuals.

### Study population

**Fig. 1:**
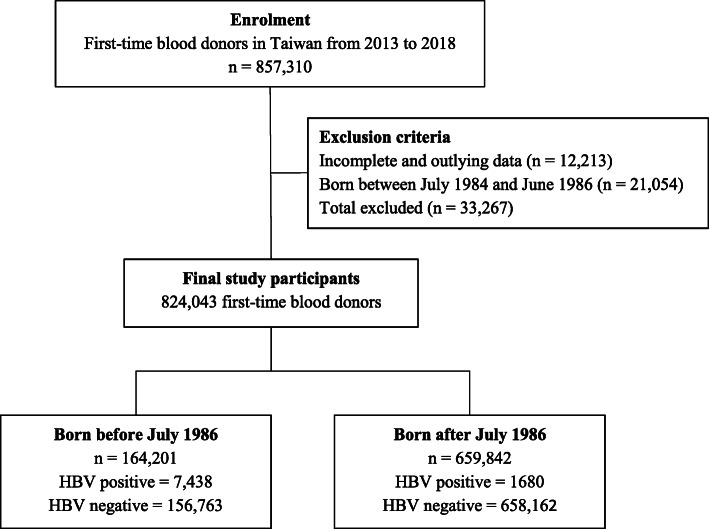
Enrolment flowchart

We recruited 857,310 first-time blood donors from 5 blood donation centers in Taiwan. We excluded donors with outlying and incomplete data (*n* = 12,213), as well as those born between July 1984 and June 1986 (*n* = 21,054). The final sample size (*n* = 824,043) consisted of 9118 HBV positive and 814,925 HBV negative individuals. Because all infants were immunized against HBV from July 1986, we divided the study population into two birth cohorts (born before and after the July 1986 HBV vaccination program). We assumed that those born before July 1986 (excluding July 1984 to June 1986) were not vaccinated at birth while those born after July 1986 were vaccinated at birth. The enrolment flowchart is shown in Fig. 1.

We also grouped participants into seven areas based on their residential addresses. The northern areas comprised Taipei, New Taipei, Keelung, and Yilan; the north-central areas included Taoyuan, Hsinchu Cities, and Miaoli; the central areas included Taichung City, Changhua, and Nantou; the central-southern areas consisted of Yunlin, Chiayi Cities and, Tainan; the southern areas comprised Kaohsiung and Pingtung, the eastern areas included Hualien and Taitung; while the island areas included Penghu, Kinmen, and Matsu. All methods were carried out following relevant guidelines and regulations. Ethical approval was obtained from the Ethical Review Board of the Taiwan Blood Services Foundation (PM-108-TC-197).

### Laboratory testing

Screening for HBV, HCV, and human immunodeficiency virus (HIV) was done by NAT using the Procleix Ultrio Plus assay (Grifols, CA, USA) on the Procleix Tigris platform (Grifols, New Hampshire, USA). All blood samples were initially screened in pools of eight. If blood samples from any pool tested positive for an infection, individual testing of all the samples in that pool was performed to check which sample was positive. Afterward, discriminatory NAT for HBV was performed to confirm the positive and negative donors. We defined an HBV infection as a positive HBV NAT. We used NAT to determine HBV status because NAT can detect an HBV infection even during the window period [26]. Moreover, it is good for detecting occult HBV infections [27].

### Statistical analysis

We compared the differences in discrete and continuous variables between the birth cohorts (born before and after July 1986) using the Chi-square and t-test, respectively. We determined the risk of HBV infection using univariate and multivariate logistic regression analysis. We also determined the interaction between birth date and sex on HBV infection using logistic regression analysis. It should be noted that we examined only the birth date/sex interaction in this study. Therefore, other interactions were not examined systematically. In the multivariate logistic regression analyses, we made adjustments for covariates including sex, age, blood type, BMI, ALT, residential area, and occupation. The odds ratios (ORs) at 95% CI were estimated. All the statistical analyses were performed using the statistical analysis system (SAS) software, version 9.4.

## Results

**Fig. 2:**
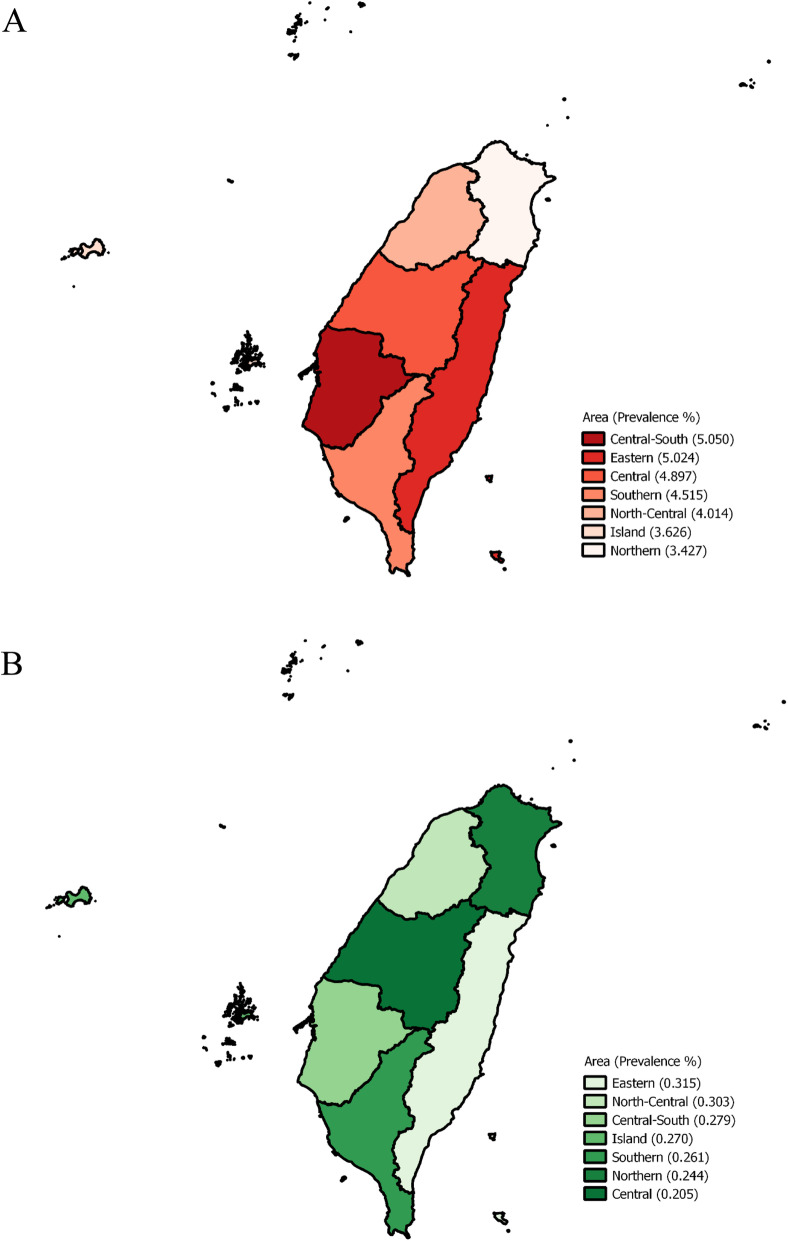
Map of Taiwan showing the prevalence of HBV infection among participants living in the various regions: **A** shows the prevalence before July 1986 while **B** shows the prevalence after July 1986

Table 1 shows the basic demographic characteristics of first-time blood donors categorized into two cohorts: born before (*n* = 164,201) and after the July 1986 HBV vaccination program (*n* = 659,842). Both cohorts had significant differences in HBV status, sex, age, BMI, ALT, residential area, and occupation (*p*-value < 0.0001). The prevalence of HBV was 4.53 and 0.25% among those born before and after July 1986, respectively. Figure 2 depicts the map of Taiwan showing the prevalence of HBV infection among participants living in various regions: 2A shows the prevalence before July 1986 while 2B shows the prevalence after July 1986.

**Table 1:** Basic information of the first-time blood donors stratified by birth date

	Born before July 1986	Born after July 1986	*p*-value
HBV NAT			< 0.001
Negative	156,763 (95.47)	658,162 (99.75)	
Positive	7438 (4.53)	1680 (0.25)	
Sex			< 0.001
Women	104,463 (63.62)	279,276 (42.32)	
Men	59,738 (36.38)	380,566 (57.68)	
Age (years)			< 0.001
17–29	1879 (1.14)	654,886 (99.25)	
30–65	162,322 (98.86)	4956 (0.75)	
Blood type			0.683
A	43,796 (26.67)	175,650 (26.62)	
B	39,811 (24.25)	159,438 (24.16)	
O	70,796 (43.12)	284,973 (43.19)	
AB	9798 (5.97)	39,781 (6.03)	
BMI (kg/m^2^)			< 0.001
≤ 24	91,484 (55.71)	462,317 (70.06)	
> 24	72,717 (44.29)	197,525 (29.94)	
ALT (U/L)	21.64 ± 17.80	17.77 ± 16.85	< 0.001
Residential area			< 0.001
Northern	57,801 (35.20)	178,238 (27.01)	
North-Central	26,669 (16.24)	99,438 (15.07)	
Central	32,150 (19.58)	144,680 (21.93)	
Central-South	21,879 (13.32)	105,406 (15.97)	
Southern	21,590 (13.15)	111,239 (16.86)	
Eastern	3387 (2.06)	17,137 (2.60)	
Island	725 (0.44)	3704 (0.56)	
Occupation			< 0.001
Student	846 (0.52)	419,940 (63.64)	
Military/civil servant/teacher	9798 (5.97)	121,172 (18.36)	
Laborer/farmer and fisherman	33,115 (20.17)	23,542 (3.57)	
Business/technician/specialist	25,511 (15.54)	14,314 (2.17)	
Service worker	34,911 (21.26)	37,933 (5.75)	
Others (including housekeepers)	60,020 (36.55)	42,941 (6.51)	

ALT was presented in mean ± standard deviation while the other variables were presented in n(%)

Table 2 shows the association of birth date and other factors with HBV infection. After adjusting for sex, age, blood type, BMI, ALT, residential area, and occupation, the risk of HBV infection was lower among participants born after July 1986 compared to those born before July 1986 (adjusted OR = 0.16, 95% CI = 0.13–0.21). Men had a higher risk of HBV infection compared to women (adjusted OR 1.41, 95% CI = 1.34–1.47). A higher risk of HBV infection was observed among individuals who were 30–65 years old compared to those who were 17–29 years old (adjusted OR = 1.66, 95% CI = 1.35–2.05) and those with a BMI > 24 kg/m^2^ compared to BMI ≤ 24 kg/m^2^ (adjusted OR = 1.08, 95% CI = 1.04–1.13). ALT was also associated with a higher risk of HBV infection (adjusted OR = 1.01, 95% CI = 1.01–1.01). With reference to the northern region, the risk of HBV was significantly higher among those who lived in the north-central, central, central-southern, southern, and eastern regions. Noteworthy, the highest risk was observed among those who lived in the eastern region. The adjusted ORs; 95% CIs were 1.16; 1.08–1.24, 1.28; 1.20–1.36, 1.39; 1.30–1.48, 1.24; 1.16–1.33, and 1.41; 1.22–1.62, corresponding to the north-central, central, central-southern, southern, and eastern regions. Based on occupation (reference group: service workers), the risk of HBV infection was significantly lower among various occupations. The lowest risk was observed among students (adjusted OR = 0.29, 95% CI = 0.26–0.33), followed by military workers/civil servants/teachers (OR = 0.69, 95% CI = 0.63–0.75).

**Table 2:** Univariate and multivariate logistic regression models showing the association of HBV infection with sex and birth date

	Crude OR	*p*-value	Adjusted OR (95% CI)	*p*-value
	(95% CI)			
Birth date				
Born before July 1986	1		1	
Born after July 1986	0.05 (0.05–0.06)	< 0.001	0.16 (0.13–0.19)	< 0.001
Sex				
Women	1		1	
Men	0.94 (0.90–0.98)	0.002	1.40 (1.34–1.47)	< 0.001
Age (years)				
17–29	1		1	
30–65	17.95 (17.02–18.92)	< 0.001	1.66 (1.35–2.05)	< 0.001
Blood type				
A	1		1	
B	0.97 (0.91–1.02)	0.227	0.96 (0.91–1.02)	0.207
O	1.01 (0.96–1.07)	0.621	1.02 (0.97–1.07)	0.526
AB	0.97 (0.88–1.06)	0.503	0.97 (0.88–1.07)	0.582
BMI (kg/m^2^)				
≤ 24	1		1	
> 24	1.99 (1.91–2.08)	< 0.001	1.08 (1.04–1.13)	< 0.001
ALT (U/L)	1.01 (1.01–1.01)	< 0.001	1.01 (1.01–1.01)	< 0.001
Residential area				
Northern	1		1	
North-Central	1.05 (0.98–1.12)	0.143	1.16 (1.08–1.24)	< 0.001
Central	1.02 (0.96–1.08)	0.471	1.28 (1.20–1.36)	< 0.001
Central-South	1.06 (0.99–1.13)	0.069	1.39 (1.30–1.48)	< 0.001
Southern	0.92 (0.86–0.98)	0.012	1.24 (1.16–1.33)	< 0.001
Eastern	1.03 (0.90–1.18)	0.667	1.41 (1.22–1.62)	< 0.001
Island	0.76 (0.55–1.05)	0.098	1.03 (0.74–1.43)	0.865
Occupation				
Service worker	1		1	
Student	0.06 (0.05–0.06)	< 0.001	0.29 (0.26–0.33)	< 0.001
Military/civil servant/teacher	0.25 (0.23–0.27)	< 0.001	0.69 (0.63–0.75)	< 0.001
Laborer/farmer and fisherman	1.43 (1.35–1.53)	< 0.001	1.03 (0.96–1.10)	0.447
Business/technician/specialist	1.01 (0.93–1.09)	0.810	0.76 (0.71–0.83)	< 0.001
Others (including housekeepers)	1.07 (1.01–1.14)	0.027	0.93 (0.88–0.99)	0.027

Table 3 illustrates the risk of HBV infection among donors born before and after the 1986 HBV vaccination program. Men had a higher risk of HBV infection than women in both birth cohorts. The adjusted ORs, 95% CIs were 1.47, 1.40–1.55 for those born before July 1986 and 1.15, 1.02–1.29 for those born after July 1986. There was a significant interaction between birth date and sex (*p*-value = 0.0067). Compared to participants aged 17–29 years, those aged 30–65 years had a higher risk of HBV infection. The adjusted ORs; 95% CIs were 1.59; 1.21–2.09 for those born before and 1.78; 1.29–2.46 for those born after July 1986. The risk was also higher among those born before July 1986 with BMI > 24 kg/m^2^ compared to BMI ≤ 24 kg/m^2^ (adjusted OR; 95% CI = 1.12; 1.07–1.18). For those born before July 1986, the highest risk of HBV was observed among those who lived in the central-southern region, followed by the eastern region (reference: the northern region). The adjusted OR, 95% CI was 1.44, 1.34–1.55 for the central-southern region and 1.42, 1.21–1.66 for the eastern region. For those born after July 1986, the highest (though not significant) risk was in the eastern (adjusted OR = 1.32, 95% CI = 0.99–1.76), followed by the north-central region (OR = 1.25, 95% CI = 1.08–1.46). Of note, the risk of HBV was significantly lower among those born after July 1986 who lived in the central region (OR = 0.83, 95% CI, 0.72–0.96). In terms of occupation (reference group: service workers), students had the lowest risk of HBV infection (adjusted OR, 95% CI = 0.45, 0.28–0.71 for those born before July 1986 and 0.25, 0.22–0.30 for those born after July 1986).

**Table 3:** Multivariate logistic regression analysis showing the risk of HBV infection stratified by birth date

	Born before July 1986	Born after July 1986
	OR (95% CI)	OR (95% CI)
Sex		
Women	1	1
Men	1.47 (1.40–1.55)	1.15 (1.02–1.29)
Age (years)		
17–29	1	1
30–65	1.59 (1.21–2.09)	1.78 (1.29–2.46)
Blood type		
A	1	1
B	0.97 (0.91–1.04)	0.94 (0.82–1.07)
O	1.03 (0.97–1.09)	0.96 (0.85–1.08)
AB	0.97 (0.87–1.08)	0.99 (0.79–1.22)
BMI (kg/m^2^)		
≤ 24	1	1
> 24	1.12 (1.07–1.18)	0.91 (0.82–1.02)
ALT (U/L)	1.01 (1.01–1.01)	1.01 (1.01–1.01)
Residential area		
Northern	1	1
North-Central	1.13 (1.05–1.22)	1.25 (1.08–1.46)
Central	1.40 (1.31–1.49)	0.83 (0.72–0.96)
Central-South	1.44 (1.34–1.55)	1.17 (1.01–1.36)
Southern	1.29 (1.20–1.39)	1.06 (0.91–1.23)
Eastern	1.42 (1.21–1.66)	1.32 (0.99–1.76)
Island	1.01 (0.68–1.49)	1.03 (0.55–1.93)
Occupation		
Service worker	1	1
Student	0.45 (0.28–0.71)	0.25 (0.22–0.30)
Military/civil servant/teacher	0.61 (0.54–0.70)	0.71 (0.60–0.85)
Laborer/farmer and fisherman	1.02 (0.95–1.09)	1.11 (0.91–1.37)
Business/technician/specialist	0.78 (0.72–0.85)	0.52 (0.38–0.73)
Others (including housekeepers)	0.98 (0.92–1.05)	0.52 (0.42–0.64)

Birth date*sex p-value = 0.0067

Table 4 shows the results of multiple logistic regression analysis using a combination of birth date and sex. Compared to the reference group (born before 1986 and the female sex), the risk of HBV infection was higher among men who were born before July 1986 (OR = 1.44, 95% CI = 1.37–1.52) but lower among both men (OR = 0.21, 95% CI = 0.17–0.26) and women (OR = 0.17, 95% CI = 0.14–0.22) born after July 1986.

**Table 4:** Odds ratios for HBV infection based on the combination of sex and birth date

	OR (95% CI)	*p*-value
Birth date and sex		
Born before July 1986, women	1	
Born before July 1986, men	1.44 (1.37–1.52)	< 0.001
Born after July 1986, women	0.17 (0.14–0.22)	< 0.001
Born after July 1986, men	0.21 (0.17–0.26)	< 0.001
Age		
17–29	1	
30–65	1.65 (1.34–2.04)	< 0.001
Blood type		
A	1	
B	0.96 (0.91–1.02)	0.206
O	1.02 (0.97–1.07)	0.527
AB	0.97 (0.88–1.07)	0.580
BMI		
≤ 24 kg/m^2^	1	
> 24 kg/m^2^	1.08 (1.03–1.13)	0.001
ALT (U/L)	1.01 (1.01–1.01)	< 0.001
Residential area		
Northern	1	
North-Central	1.16 (1.08–1.24)	< 0.001
Central	1.28 (1.21–1.36)	< 0.001
Central-South	1.39 (1.30–1.49)	< 0.001
Southern	1.25 (1.16–1.33)	< 0.001
Eastern	1.41 (1.22–1.61)	< 0.001
Island	1.03 (0.74–1.43)	0.872
Occupation		
Service worker	1	
Student	0.29 (0.26–0.32)	< 0.001
Military/civil servant/teacher	0.70 (0.64–0.77)	< 0.001
Laborer/farmer and fisherman	1.02 (0.96–1.09)	0.505
Business/technician/specialist	0.76 (0.71–0.83)	< 0.001
Others (including housekeepers)	0.94 (0.88–0.99)	0.044

## Discussion

In the current study, the 6-year prevalence of HBV (based on NAT) among first-time blood donors was 1.1%. We observed a decrease in HBV infection from 4.53% among first-time donors born before the July 1986 vaccination program to 0.25% among those born after the program. Statistics from the Taiwan Blood Services Foundation showed a decrease in the percentage of first-time donors (from 15.66% in 2010 to 12.8% in 2019). Moreover, the average blood donation rate between 2013 and 2018 was just about 7.5% [28]. Because some eligible blood donors are aging, there is a need to recruit more first-time donors to ensure the sustainability of blood supply [29]. The decrease in HBV infection after the July 1986 vaccination program observed in the current study reflects an increase in the percentage of eligible first-time donors as well as first-time donors eligible for subsequent blood donations.

HBV is a major cause of hepatocellular carcinoma [1]. To decrease the risk of HBV infection, the Taiwan government and health care workers implemented the universal HBV vaccination program in 1984 [30]. It should be noted that Taiwan was the first country to launch the HBV vaccination program in the world [11]. This preventive effort effectively reduced the incidence of HBV in Taiwan [31] and converted the island from a hyper- to a low-endemic region [14]. Hepatitis B vaccination in infants also reduced hepatocellular carcinoma risk in both Taiwanese children and adults [32].

A previous study reported the prevalence of 4.1% for acute and 1.4% for chronic HBV infection in Taiwanese adults who were fully vaccinated (completed a four-dose plasma-derived HBV vaccination schedule) during their infancy [33]. In the current study, the prevalence of HBV among the supposed vaccinated group (born after July 1986) was 0.25%. Some of the reasons for this difference could be because those with a personal history of surgery or blood transfusion were not eligible for blood donation and were, therefore, not included in our final analyses. Moreover, all high-risk individuals (those who had sex with viral hepatitis patients or were exposed to blood/body fluids) and those who had a diagnosis of viral hepatitis in the past year were not eligible for blood donation. NSAIDs have anti-inflammatory properties and could reduce the risk of chronic neoplastic progression [34]. Moreover, they could suppress viral replication through the inhibition of prostaglandin E2 (PGE2) [35]. Taking such medications could mean that an individual might have been previously exposed to HBV and was, therefore, not eligible for blood donation.

The proportion of men with positive HBV NAT before and after the implementation of the HBV vaccination program was higher than women. These findings are congruent to those previously reported among Taiwanese after a follow-up of over 18 years [36]. Serum levels of HBsAg are also higher in men than women [37, 38]. Moreover, after HBV vaccination, men have higher titers of anti-HBs antibodies than women [36]. The sex disparity in HBV infection is shaped by genetic factors and mainly regulated by sex hormones [39]. The main male hormone (androgen) has immune-suppressing effects while the female hormone (estrogen) has immune-enhancing properties. Therefore, the higher risk in men and lower risk in women could be attributed to the abundance of androgens and estrogens which could respectively suppress and promote immune responses to infections [39–41]. The X chromosome is believed to be enriched with genes that have immune responses [42, 43]. Therefore, the lower risk of HBV in women could also be attributed to the presence of two X chromosomes in women. Furthermore, a variant of the HBV receptor gene, sodium taurocholate co-transporting polypeptide (NTCP) was associated with a lower risk of HBV infection among Taiwanese women [44].

HBV infection was higher among older first-time donors compared to their younger counterparts. This could be because older individuals may have not been vaccinated at birth. Besides, the deterioration of the immune system termed, immunosenescence is related to aging [45]. As people get older, their immune systems become compromised making them vulnerable to infections.

Compared to normal-weight individuals, obese people have a higher likelihood of bacterial, viral, and fungal infections [46]. Moreover, poor response to hepatitis B vaccination is common among obese individuals with a compromised immune system [46]. Loss of response to HBV immunization is also related to obesity [47]. One reason could be due to leptin which is produced by fat cells. Abnormal levels of this hormone could induce systemic and B cell-intrinsic inflammation, weaken T cell responses, and impair the division and proliferation of lymphocytes in obese people, thereby increasing their risk of HBV infection [47].

Determination of blood groups is very important before a blood transfusion because blood groups are associated with certain diseases [48]. HBV infection was not associated with blood group in the current study. Previous findings on the association of blood groups with HBV have not been coherent. For instance, in a meta-analysis, blood group B was associated with a lower risk of HBV infection while blood group O was not significantly associated with HBV infection among Asians [49]. In another study, blood group O was associated with a lower risk of HBV infection among Iranian blood donors [50]. On the contrary, blood group O was associated with a higher risk of HBV among Chinese [23]. The discrepancy between our study and previous studies could be due to different prevalence rates, demographic characteristics, and different ratios of blood types. Further investigations of this relationship are warranted.

In this study, we categorized hairdressers and healthcare workers as service workers and used them as the reference group because they are high-risk individuals. Besides, the sample sizes in this group were comparable in both birth cohorts (before and after 1986). The risk of HBV is higher among hairdressers [51] and healthcare workers [52], probably because of high exposure to sharp objects [53].

In our study, living in eastern Taiwan was associated with the highest risk of HBV in most scenarios. In a previous study, the prevalence of HBV was higher among the indigenous people than the other Taiwanese populations [54]. Most indigenous people live in the eastern part of the country like Taitung and Hualien which have insufficient medical facilities compared to other parts [55]. Interestingly, we found that living in Central Taiwan was associated with a higher risk of HBV among those who were born before July 1986. However, living in the same area was associated with a lower risk among individuals who were born after July 1989. We cannot comprehensively explain the mechanism underlying the observed associations and therefore, recommend further investigations to clarify our findings.

The strength of this study is its relatively large sample size. However, there are some limitations. First, even though the prevalence of hepatitis B infection in smokers is higher than in nonsmokers [56], we did determine the association of smoking with HBV because the health questionnaires did not contain information on cigarette smoking. Second, marital status, education level, and the family history of HBV are some major risk factors of HBV infection [57]. To follow the “Personal Information Protection Act“, the marital status and education level were not included in the blood donor registration form and therefore, these factors were not evaluated. The health questionnaire did not also have information on the family history of HBV. Third, it is reported that about 77% of HBsAg-positive individuals under 30 years were born to HBsAg-positive mothers [58]. However, our questionnaires did not contain information on participants’ mothers’ HBV status or vaccination history. Therefore, we could not determine what proportion of the donors were born to HBsAg-positive mothers. Lastly, we did not know exactly how many people were vaccinated from July 1986. However, it has been shown that at least 90% of children in Taiwan born after 1986 received vaccination at birth [59].

## Conclusions

In conclusion, the prevalence of HBV among first-time blood donors decreased from 4.2% before the July 1986 HBV vaccination program to 0.25% after the program, suggesting that more first-time blood donors could be eligible for subsequent blood donations. The risk of HBV was lower among those who were born after compared to those who were born before July 1986. In both cohorts, the risk was high in men relative to women. The protective effect among those born after July 1986 was higher in women than men. Age ≥ 30 years, BMI ≥ 24 kg/m^2^, living in the eastern part of the country, and occupations other than students were associated with a higher risk of HBV. To avoid HBV infection, the population should be educated on the importance of taking appropriate preventive measures especially vaccination.

## Data Availability

The data that support the findings of this study are available from the Taiwan Blood Services Foundation (TBSF) but restrictions apply to the availability of these data, which were used under license for the current study, and so are not publicly available. Data are however available from Professor Yung Po Liaw (Email address: Liawyp@csmu.edu.tw, Tel: + 886424730022 ext. 12102; fax: + 886423248179) upon reasonable request and with permission of Taiwan Blood Services Foundation (TBSF).

## Abbreviations

HBV: Hepatitis B virus
OR: Odds ratio
CI: Confidence interval
HCC: Hepatocellular carcinoma
HBsAg: HBV surface antigen
NAT: Nucleic amplification test
BMI: Body mass index
Hb: Hemoglobin
TTVI: Transfusion-transmitted viral infection
HCV: Hepatitis C
ALT: Alanine aminotransferase
